# A Text-Book of Medical Treatment of Diseases and Symptoms for the Use of Students and Practitioners of Medicine

**Published:** 1900-09

**Authors:** 


### Books and Magazines.

A TEXT-BOOK OF MEDICAL TREATMENT OF DISEASES AND SYMPTOMS FOR THE USE OF STUDENTS AND PRACTITIONERS OF MEDICINE.—By Nestor Tirard, M. D., F. R. C. P., Professor of Principles and Practice of Medicine, King's College, London. Adapted to the U. S. Pharmacopœia by E. Quin Thornton, M. D., of Jefferson Medical College, Philadelphia. In one octavo volume of 624 pages. Just ready. Cloth, $4.00 net. Lea Brothers & Co., Philadelphia and New York.

There should be a demand in this country for a good book on treatment. The average text-book on practice dismisses the treatment with a few lines. At best, in many of the works, the treatment is dealt with only in a general way, and nothing of detail and specific knowledge are given. This work is devoted directly to treatment, giving in clear and specific terms the most modern and approved agencies and methods. It is an English work, as shown by the title, but its full therapeutical directions and prescriptions have been revised to conform to the United State Pharmacopœia.

